# Supplementary material for: The Combination of Molecular Adjuvant CCL35.2 and DNA Vaccine Significantly Enhances the Immune Protection of Carassius auratus gibelio against CyHV-2 Infection
Source: Vaccines (Basel). 2020 Oct 1;8(4):567. doi: 10.3390/vaccines8040567 (PMC7712643; doi:10.3390/vaccines8040567)
Supplement: Supplementary file 1 [file vaccines-08-00567-s001.pdf]

Appendix

Table S1 Primer sequences in this study.

Gene name	Primer name	Primer sequence (5'-3')	Amplicon size (nt)
ORF25	A036F	TCGAGGATCCGCCACCATGGGGTTCCAACGGTGTCTG	1818
	A036R	TCGAGAATTCTCAAGCGTAATCTGGAACATCGTATGGGTAAGAGGGCAACTTCACGTAC	
CCL35.2	A037F	TCGAGGATCCGCCACCATGTCTGCTTCTCGCTTCGTC	324
	A037R	TCGAGAATTCTTAAGCGTAATCTGGAACATCGTATGGGTACAAGGTCCCTTGGCTTCCG	
IL-1β	A052F	ACTGGAGATGTCTTCGCATCC	119
	A052R	GCAGCGCATCTTCACAAATCT	
IL-2	A053F	GACCACAAAGGTAGACCCATCC	212
	A053R	GAGGTTTGTGCGGAATGGAC	
IFN-γ2	A054F	GACTGCGCAACACATGATGG	112
	A054R	CTCGTCGATGCTCTTGTCCA	
Viperin	A055F	AAGTCATAGGTCGAGGTCAGGG	207
	A055R	CGTTTTCACCTTCAATTAGCAGA	
IgM	A056F	GTCAATCTTCGGCTTGTCTCA	179
	A056R	GGGTATAATCTCCATCGGGTC	
TK	A057F	ACAGCGGAGCAAAATACCCA	236
	A057R	TTTGTTGGCGACGGGGATAA	
	A040F*	ATGGCTTTTCTGGAGTTGGT	324
	A040R*	GCACCCACGATAACATACTT	
β-actin	A058F	ACCCCATTGAGCACGGTATT	235
	A058R	CGACCAGAAGCGTACAGAGA	

* The primers were used in virus detection in serum-incubation-infection experiments.

Table S2 Statistical results of histological lesions in gill, spleen and trunk kidney of gibel carp on day 7 post CyHV-2 infection.

−, none; +, mild; ++, moderate; +++, severe. n = 3.

	Gill		Spleen		Trunk kidney	
Lesion	SEC	SBLC	Vacuolization	Karyorrhexis	Hypertrophied nuclei	Vacuolization
Control	-	-	-	-	-	-
CCL35.2/ORF25	-	+	+	-	+	-
ORF25	-	+	+	+	+	+
CCL35.2/Vaccine	-	+ +	++	+	-	+
Vaccine	+	+ +	+ +	+ +	+ +	+ + +
CCL35.2	+ + +	+ + +	+ +	+ +	+ + +	+ + +
pcDNA3.1-neo	+ + +	+ + +	+ +	+ ++	+ + +	+ + +
PBS	+ + +	+ + +	+ + +	+ + +	+ + +	+ + +

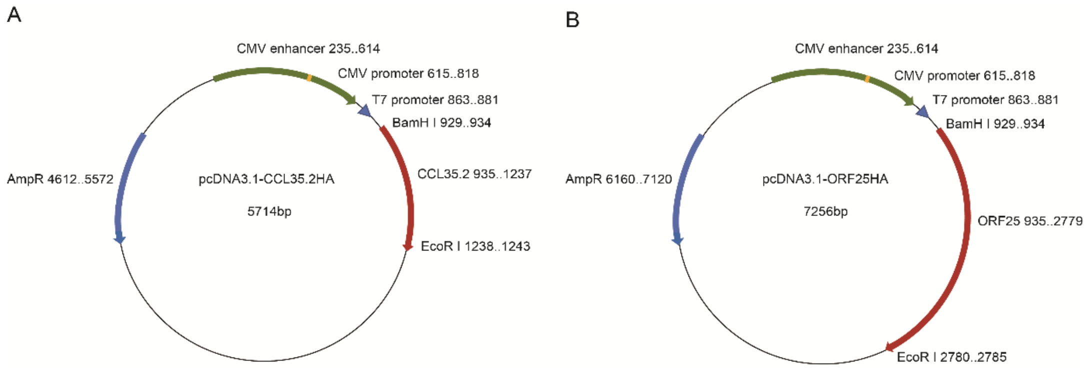

Figure S1 The plasmid maps of pcDNA3.1-CCL35.2HA and pcDNA3.1-ORF25HA. BamH I with kozak sequence (GCCACC) and EcoR I restriction digestion sites were added to the forward and reverse primers respectively.
